# Glycerophosphatidylcholine PC(36:1) absence and 3′-phosphoadenylate (pAp) accumulation are hallmarks of the human glioma metabolome

**DOI:** 10.1038/s41598-018-32847-8

**Published:** 2018-10-03

**Authors:** Wenchen Li, Hongmei Jia, Qi Li, Jiayue Cui, Ri Li, Zhongmei Zou, Xinyu Hong

**Affiliations:** 1 Department of Neurosurgical Oncology, The First Hospital of Jilin University, Changchun, PC:130021 China; 2 Institute of Medicinal Plant Development, Chinese Academy of Medical Sciences and Peking Union Medical College, Beijing, PC:100191 China; 3 Core Laboratory for Clinical Medical Research, Beijing Tian Tan Hospital, Capital Medical University, Beijing, PC:100050 China; 4 Department of Histology and Embryology of Basic Medicine College, Jilin University, Changchun, PC:130021 China

## Abstract

Glioma is the most prevalent malignant brain tumor. A comprehensive analysis of the glioma metabolome is still lacking. This study aims to explore new special metabolites in glioma tissues. A non-targeted human glioma metabolomics was performed by UPLC-Q-TOF/MS. The gene expressions of 18 enzymes associated with 3’-phosphoadenylate (pAp) metabolism was examined by qRT-PCR. Those enzymes cover the primary metabolic pathway of pAp. We identified 15 new metabolites (13 lipids and 2 nucleotides) that were significantly different between the glioma and control tissues. Glycerophosphatidylcholine [PC(36:1)] content was high and pAp content was significantly low in the control brain (p  < 0.01). In glioma tissues, PC(36:1) was not detected and pAp content was significantly increased. The gene expressions of 3′-nucleotidases (Inositol monophosphatase (*IMPAD-1*) and 3′(2′),5′-bisphosphate nucleotidase 1(*BPNT-1*)) were dramatically down-regulated. Meanwhile, the gene expression of 8 sulfotransferases *(SULT)*, 2 phosphoadenosine phosphosulfate synthases (*PAPSS-1 and PAPSS-2*) and L-aminoadipate-semialdehyde dehydrogenase-phosphopante-theinyl transferase (*AASDHPPT*) were up-regulated. PC(36:1) absence and pAp accumulation are the most noticeable metabolic aberration in glioma. The dramatic down-regulation of *IMPAD-1* and *BPNT-1* are the primary cause for pAp dramatic accumulation. Our findings suggest that differential metabolites discovered in glioma could be used as potentially novel therapeutic targets or diagnostic biomarkers and that abnormal metabolism of lipids and nucleotides play roles in the pathogenesis of glioma.

## Introduction

Glioma is the most prevalent primary brain tumor that arises from astrocytes or oligodendrocytes or mixed glial populations^1^. Gliomas are classified by the World Health Organization (WHO) into four grades^2^. WHO grades I and II are considered to be low grade gliomas while WHO grades III and IV are high grade gliomas^3^. More than 50% of the glioma patients are presented with glioblastoma (WHO grade IV)^4^.

Metabolomics is the systemic study of metabolites generated during different biological processes in cells, tissues, and organisms. Owing to innovative developments in informatics and analytical technologies, metabolomics has great potential in discovering clinical biomarkers and treatment targets, as well as elucidating previously unknown mechanisms^5^. The advances in the technology platforms led to a renaissance of metabolomics research in recent years^6,7^.

The past decade has witnessed remarkable progress in elucidating the genetic causes and oncogenesis mechanism for some of glioma^8^. 2-hydroxyglutarate (2-HG) is a specific oncometabolite that has been identified as a putative biomarker in isocitrate dehydrogenase 1 and 2 (IDH1 and IDH2) mutant gliomas^9^. A recent study raised the possibility that glutamate dehydrogenase2(GDH2)specific inhibition may serve as a viable therapeutic strategy for gliomas with IDH mutations^10^.

To date, apart from 2-HG, many metabolites associated with glioma have been reported^11–14^. Nevertheless, the number of significant metabolites discovered in glioma tissues is limited. Therefore, in this study, we prepared the polar and non-polar solvent extracts obtained from glioma and healthy control tissues and performed a non-targeted tissue metabolomics analysis. Non-targeted analysis of biomolecules still is an important trend in the field of “omics” including proteomics, genomics, transcriptomics and metabolomics^15^. In order to get unbiased metabolite profiling of glioma, we used the analysis procedure of discovery set and validation set^16^.

We used UPLC-Q-TOF/MS to compare the metabolomics profiling of glioma tissues and control brain parenchyma. Further, we examined the gene expressions of 18 enzymes associated with pAp metabolism by quantitative real-time polymerase chain reaction (qRT-PCR). These 18 enzymes cover primary metabolic pathways of pAp.

## Results

### Metabolomics profiling of glioma and control tissue in the discovery set

**Figure 1:**
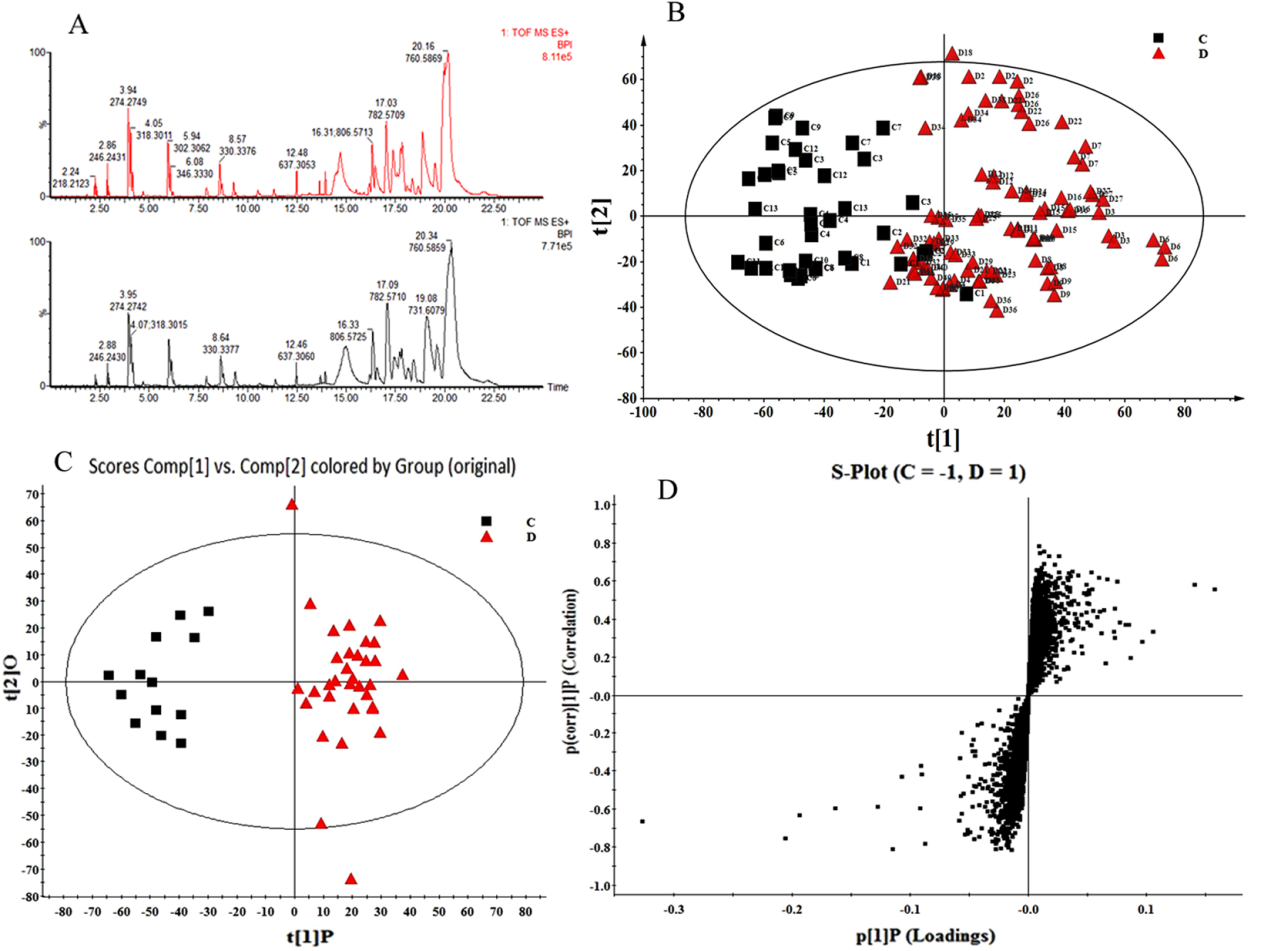
Analysis of PCA and OPLS-DA in discovery set. (**A**) Typical UPLC-Q/TOF MS base peak intensity (BPI) chromatogram of glioma and control samples in positive ion mode; (**B**) PCA analysis based on UPLC-Q-TOF-MS data obtained from the glioma and control samples in the discovery set. PCA score plot (R2X = 0.821, Q2 (cum) = 0.967); (**C**) OPLS-DA score plot (R2X = 0.868, R2Y = 0.996, Q2 (cum) = 0.941); (**D**) S-plot.

The discovery set included 13 control specimens and 33 glioma specimens. Analysis of PCA and OPLS-DA in discovery set is shown in Fig. 1.

The representative base peak intensity chromatograms and PCA analysis of the glioma and control tissues are shown in Fig. 1A,B. In order to explore the inherent grouping between glioma patients and healthy control subjects, PCA was used to map the samples based on their spectral profile without previous knowledge of the class. Score plot demonstrated that the metabolic profiles of glioma patients deviated from the control samples suggesting that significant biochemical changes must have occurred in glioma tissues. Next, an OPLS-DA model was employed to refine the separation results obtained by PCA. Indeed, the score plot resulting from the OPLS-DA model showed a superior separation between the glioma and control tissues (Fig. 1C). The corresponding S-plot indicated that the differential metabolites with VIP value ≥ 1 were responsible for discriminating glioma and control samples (Fig. 1D).

Next, analysis of the accurate molecular weights and MS^E^ spectra through HMDB (http://www.hmdb.ca/) and KEGG (http://www.genome.jp/kegg/) within a mass difference <5 ppm revealed 15 significantly variable metabolites between glioma and control samples (Table 1).

**Table 1:** Differential metabolites of glioma specimens detected by UPLC/QTOF-MS.

No	Metabolites Common name	Retention Time	Molecular Mass	Formula	Trend (Tvs C)	VIP	Class
1	pAp/ 3′-Phosphoadenylate	9.55	427.2011	C_10_H_15_N_5_O_10_P_2_	↑^**^	7.12	Purine Nucleotides
2	Appr > p/ ADP-ribose 1′′-2′′ cyclic phosphate	2.19	621.2804	C_15_H_22_N_5_O_16_P_3_	↑^**^	4.65	Purine Nucleotides
3	2-Hexaprenyl-6-methoxyphenol	2.11	532.8393	C_37_H_56_O_2_	↑ ^**^	5.25	Prenol lipids
4	DG(33:3)/ DG(15:0/18:3(9Z,12Z,15Z)/0:0)	20.80	576.8904	C_36_H_64_O_5_	↑^**^	8.03	Glycerolipids
5	CE(15:0)/ 15:0 cholesterol ester	6.01	611.0358	C_42_H_74_O_2_	↑^**^	7.32	Cholesterol ester
6	PC(36:1)/ PC(18:1(11Z)/18:0)	14.88	788.1293	C_44_H_86_NO_8_P	↓^**^	6.06	Glycerophosphatidylcholine
7	PC(36:3)/ PC(22:2(13Z,16Z)/14:1(9Z))	18.04	784.0975	C_44_H_82_NO_8_P	↑^**^	7.01	Glycerophosphatidylcholine
8	PC(38:6)/ PC(20:5(5Z,8Z,11Z,14Z,17Z)/P-18:1(11Z))	16.78	790.1037	C_46_H_80_NO_7_P	↑^**^	7.59	Glycerophosphatidylcholine
9	PC(38:4)/ PC(P-18:0/20:4(5Z,8Z,11Z,14Z))	20.39	794.1354	C_46_H_84_NO_7_P	↑^**^	8.31	Glycerophosphatidylcholine
10	LysoPC(O-18:0)	15.44	509.6997	C_26_H_56_NO_6_P	↑^**^	8.74	Lyso-glycerophosphatidylchoine
11	LysoPE(18:3)/ LysoPE(18:3(6Z,9Z,12Z)/0:0)	10.52	475.5558	C_23_H_42_NO_7_P	↑^**^	7.87	Lyso-glycerophosphatidylethanolamine
12	LysoPE(20:3)/ LysoPE(20:3(8Z,11Z,14Z)/0:0)	12.85	503.6090	C_25_H_46_NO_7_P	↑^**^	7.19	Lyso-glycerophosphatidylethanolamine
13	LysoPE(18:0)/ LysoPE(0:0/18:0)	13.94	481.6035	C_23_H_48_NO_7_P	↑^**^	6.95	Lyso-glycerophosphatidylethanolamine
14	Ceramide(d18:1/16:0)/C16 Cer	16.36	537.9007	C_34_H_67_NO_3_	↑^**^	6.68	Sphingolipids
15	Sphingomyelin SM(d18:0/12:0)/C12 sphingomyelin	16.86	650.9536	C_35_H_75_N_2_O_6_P	↑^**^	7.56	Sphingomyelin(with phosphocholine)

Foot notation: 1. (T VS C) means tumor vs control. 2. VIP: variable importance in the project. **p ≤ 0.01.

**Figure 2:**
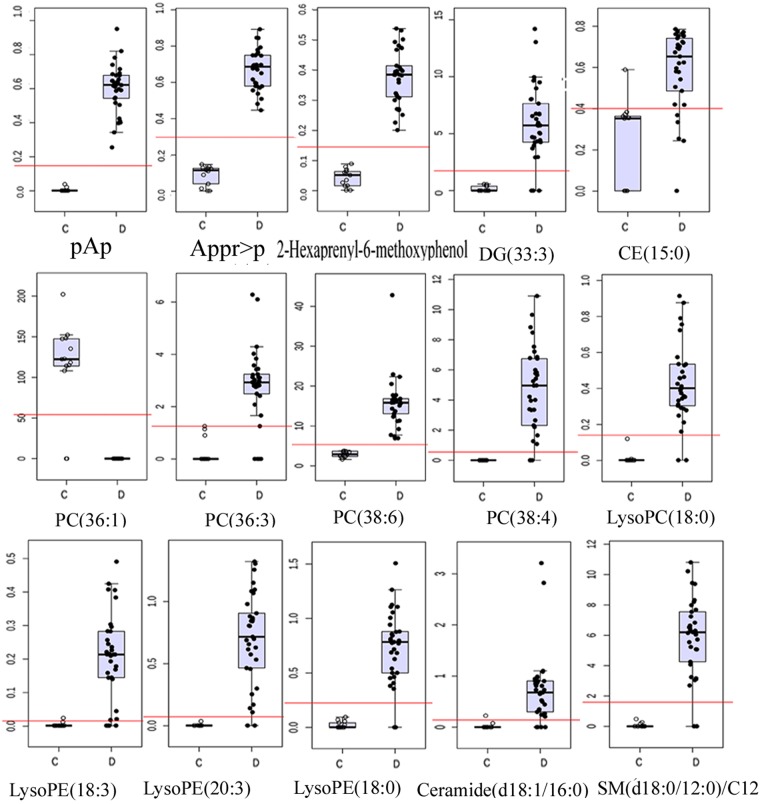
Box plots of the levels of 15 discriminating metabolites in the discovery set.C: Control group; D: Glioma group.

The relative abundances of the identified metabolites in the discovery set were shown in Fig. 2.

Among the discovered metabolites, we observed two distinct classes: purine nucleotide(pAp and Appr > p) and lipids (glycerolipids, sterol lipids, prenol lipids, phospholipids and sphingolipids).

Our results demonstrated that Glycerophosphatidylcholine PC(36:1) was not detected in the glioma tissues, but its level was significantly high in the control specimens (p  < 0.01, Fig. 2, Table 1). Compared to the control group, the levels of glycerophosphatidylcholine [PC(38:4), PC(38:6), PC(36:3)] lyso- glycerophosphatidylcholine [lysoPC(18:0)] and all Lyso-glycerophosphatidylethanolamine [lysoPE(18:3), lysoPE(20:3), lysoPE(18:0) and lysoPE (34:1)], Diacylglycerol [DG(33:3)], phenolic lipid(2-Hexaprenyl-6-methoxyphenol), cholesterol ester [CE (15:0)] and sphingolipids [SM(d18:0/12:0) and Ceramide (d18:1/16:0)] were significantly higher in the glioma tissues (p < 0.01, Fig. 2, Table 1). Similarly, the levels of pAp and ADP-ribose 1″-2″ cyclic phosphate(Appr > p)were obviously higher in glioma tissues (p < 0.01, Fig. 2, Table 1).

### Metabolomics profiling of glioma and control tissue in the validation set

**Figure 3:**
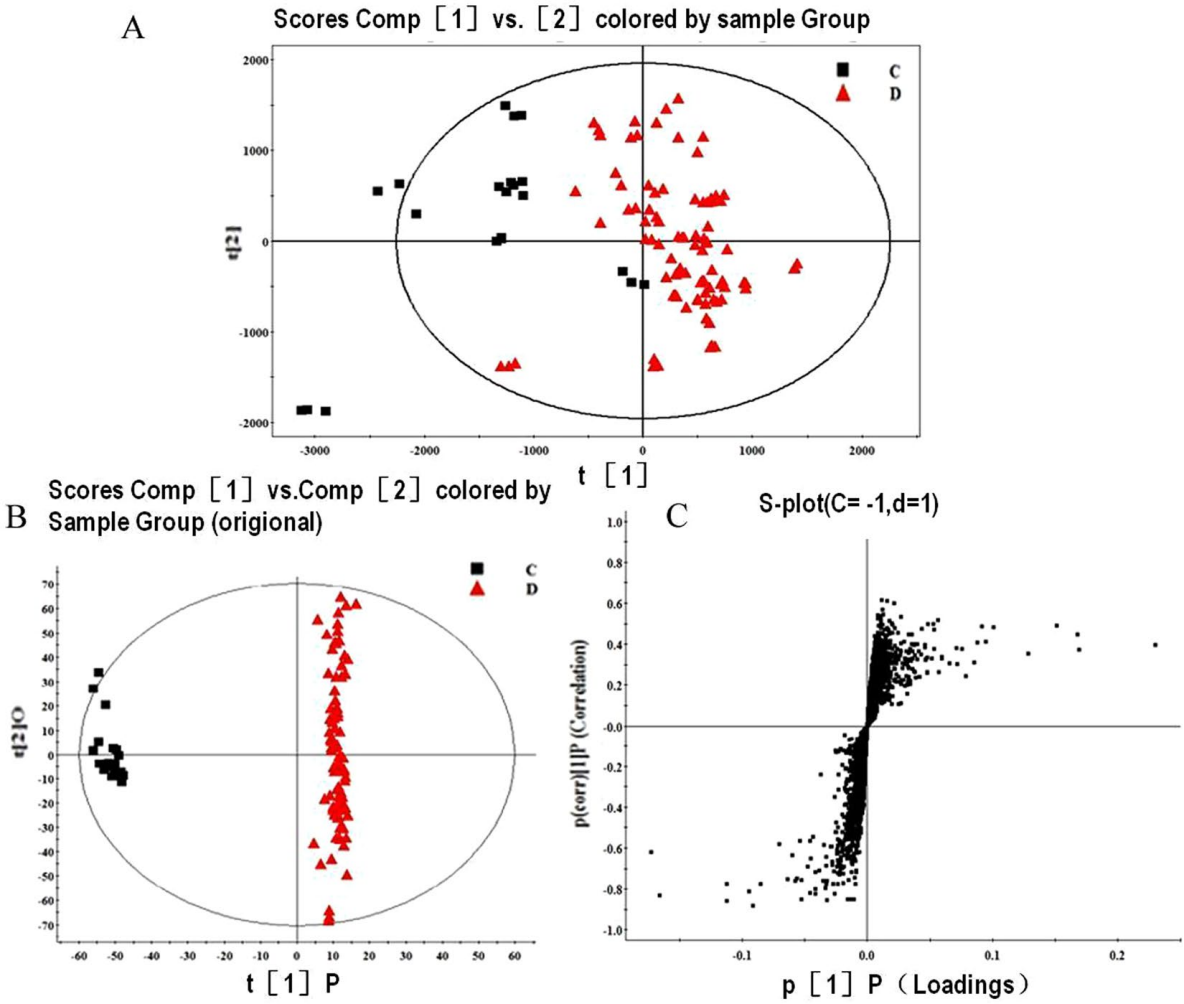
Analysis of PCA and OPLS in validation set (**A**). PCA score plot analysis (R2X = 0.856, Q2 (cum) = 0.804); (**B**) OPLS-DA Score plot analysis (R2X = 0.766, R2Y = 0.985, Q2(cum) = 0.832) of the results obtained from the glioma tissues and control brain parenchyma in the validation set; (**C**) S-plot of the validation set.

To validate the observed findings in discovery set, 7 control brain parenchyma samples, 26 glioma specimens were collected and analyzed under the same analytical procedures (Fig. 3).

The relative content of the 15 distinctive metabolites in the validation set is shown in Table 2. In addition, statistical analysis demonstrated that the content difference of each metabolite between the gliomas (both WHO grade III-IV gliomas and the WHO grade II) and control specimens was significant (P < 0.01, Table 2). On the other hand, no statistically significant difference was observed between the WHO grades II and III-IV gliomas (P > 0.05).

**Table 2:** The relative content of the 15 distinctive metabolites in the validation set. Median(IQR)/mean ± SD. *comparison between control tissues and WHO grades I–II gliomas; ^#^comparison between the control tissues and WHO grades III–IV gliomas, the p of * and ^#^ < 0.05. p value in right column means the statistical differences among 3 groups (control, WHO grades I and II and WHO grades III and IV group).

matabolite	Control	WHO grades I and II	WHO grades III and IV	*P* value
pAp	0.001 (0.000, 0.010)	0.610 (0.503, 0.798)*	0.650 (0.533, 0.743)^#^	<0.001
Appr > p	0.094 ± 0.019	0.771 ± 0.144*	0.680 ± 0.207^#^	<0.001
2-Hexaprenyl-6-methoxyphenol	0.048 ± 0.021	0.350 ± 0.096*	0.363 ± 0.126^#^	<0.001
DG(33:3)	0.110 (0.000, 0.521)	8.121 (5.023, 19.581)*	6.546 (4.714, 8.981)^#^	<0.001
CE(15:0)	0.218 ± 0.069	1.019 ± 1.127*	0.615 ± 0.209^#^	0.031
PC(36:1)	110.489 ± 14.283	0.000 ± 0.000*	0.000 ± 0.000^#^	<0.001
PC(36:3)	0.220 (0.000, 0.341)	6.008 (4.426, 6.610)*	3.019 (2.570, 3.543)^#^	<0.001
PC(38:6)	2.855 (2.457, 3.748)	3.526 (2.755, 4.448)*	16.551 (15.235, 17.338)^#^	<0.001
PC(38:4)	0.000 ± 0.000	7.712 ± 2.488*	3.998 ± 2.761^#^	<0.001
LysoPC(18:0)	0.000 (0.000, 0.003)	0.316 (0.289, 0.426)*	0.396 (0.356, 0.454)^#^	<0.001
LysoPE(18:3)	0.000 (0.000, 0.002)	0.253 (0.218,0. 374)*	0.203 (0.117, 0.285)^#^	0.001
LysoPE(20:3)	0.000 (0.000, 0.004)	0.660 (0.556, 0.828)*	0.712 (0.533, 0.881)^#^	<0.001
LysoPE(18:0)	0.017 ± 0.020	0.741 ± 0.072*	0.716 ± 0.114^#^	<0.001
Ceramide(d18:1/16:0)	0.019 (0.000, 0.031)	0.785 (0.500, 0.847)*	0.798 (0.639, 0.857)^#^	<0.001
SM(d18:0/12:0)/C12	0.025 (0.000, 0.102)	8.191 (3.103, 10.808)	6.303 (3.166, 6.832)^#^	0.009

Taken together, results obtained from both discovery and validation sets demonstrated that the PC(36:1) was an abundant component of the control brain parenchyma, while pAp content was significantly low. In contrast, PC(36:1) was not detected in glioma tissues and the pAp content was remarkably increased. The PC(36:1) absence and pAp accumulation were the most obvious characteristics in glioma metabolomes. Secondly, the abundance of PC(38:4) mass was relatively rich in glioma, but the variation degree of PC(38:4) was lower than that of PC(36:1) (Fig. 2, Table 2).

### Gene expression of enzymes associated with pAp metabolism

Metabolomics profiling of glioma tissues demonstrated a notably higher pAp content (Fig. 2, Table 2). pAp is a toxic by-product^17^. In order to illuminate the mechanisms that led to the accumulation of pAp within glioma tissues, 18 enzyme genes related to pAp metabolism were analyzed by qRT-PCR. A comprehensive list of the analyzed genes was mentioned in Table 3.

**Table 3:** The comprehensive information list of 18 enzymes related to pAp metabolism.

NO	Gene name	Protein name	Catalytic activity	Gene location
1	*AASD- HPPT*	L-aminoadipate-semialdehyde dehydrogenase-phosphopante- theinyl transferase	CoA-[4′-phosphopantetheine] + apo-ACP = pAp + holo-ACP	11q22.3
2	*NDST2*	Bifunctional heparan sulfate N-deacetylase/N-sulfotransferase 2	PAPS + [heparan sulfate]-glucosamine = pAp + [heparan sulfate]-N-sulfoglucosamine (glucosamine 3-sulfate) catalyzes both the N-deacetylation and the N-sulfation of glucosamine (GlcNAc)	10q22.2
3	*NDST4*	Bifunctional heparan sulfate N-deacetylase/N-sulfotransferase 4	same as above	4q26
4	*HS3ST2*	Heparan sulfate glucosamine 3-O-sulfotransferase 2	PAPS + [heparan sulfate]-glucosamine = pAp + [heparan sulfate]-glucosamine 3-sulfate	16p12.2
5	*HS3ST4*	Heparan sulfate glucosamine 3-O-sulfotransferase 4	Same as above	16p12.1
6	*HS3ST5*	Heparan sulfate glucosamine 3-O-sulfotransferase 5	Same as above	6q21- q22.1
7	*HS6ST3*	Heparansulfate glucosamine 6-O-sulfotransferase 3	PAPS + [heparan sulfate]-glucosamine = pAp + [heparan sulfate]-glucosamine 6-sulfate	13q32.1
8	*CHST3*	Carbohydrate sulfotransferase 3	PAPS + chondroitin = pAp + chondroitin 6′-sulfate	10q22.1
9	*CHST7*	Carbohydrate sulfotransferase 7	Same as above	Xp11.3
10	*CHST12*	Carbohydrate sulfotransferase 12	PAPS + chondroitin = pAp + chondroitin 4′-sulfate	7p22.3
11	*CHST15*	Carbohydrate sulfotransferase 15	PAPS + [dermatan/chondroitin]-4-O-sulfo-N-acetylgalactosamine = pAp + [dermatan/chondroitin]-4,6-di-O-sulfo-N- acetyl-D-galactosamine	10q26.13
12	*TPST1*	Protein-tyrosine sulfotransferase 1	PAPS + protein tyrosine = pAp + protein tyrosine- O-sulfate	7q11.21
13	*TPST2*	Protein-tyrosine sulfotransferase 2	Same as above	22q12.1
14	*GAL3-* *ST1*	Galactosylceramide sulfotransferase	PAPS + a galactosylceramide = pAp + a galactosyl ceramidesulfate PAPS + monogalactosylalkylacylglycerol = pAp + monogalactosylalkylacylglycerol sulfate	22q12.2
15	*PAPSS1*	Bifunctional PAPS synthase 1	ATP + sulfate = diphosphate + AMP-SO_3_^−^ (APS) ATP + AMP-SO_3_^−^ (APS) = ADP + PAPS	4q25
16	*PAPSS2*	Bifunctional PAPS synthase 2	Same as above	10q23.2- q23.31
17	*IMPAD1*	Inositol monophosphatase 3	Myo-inositol phosphate + H_2_O = myo-inositol + phosphate pAp + H_2_O = 5′-AMP + phosphate	8q12.1
18	*BPNT1*	3′(2′),5′-bisphosphate nucleotidase 1	pAp + H2O = 5′-AMP + phosphate	1q41

Foot notation: CoA: Coenzyme A; ACP: acylcarrier protein; PAPS: Phosphoadenosine phosphosulfate; pAp: 3′-Phosphoadenylate; APS: Adenosine-5′-phosphosulfate. Data was compiled according to HMDB 2018.

**Figure 4:**
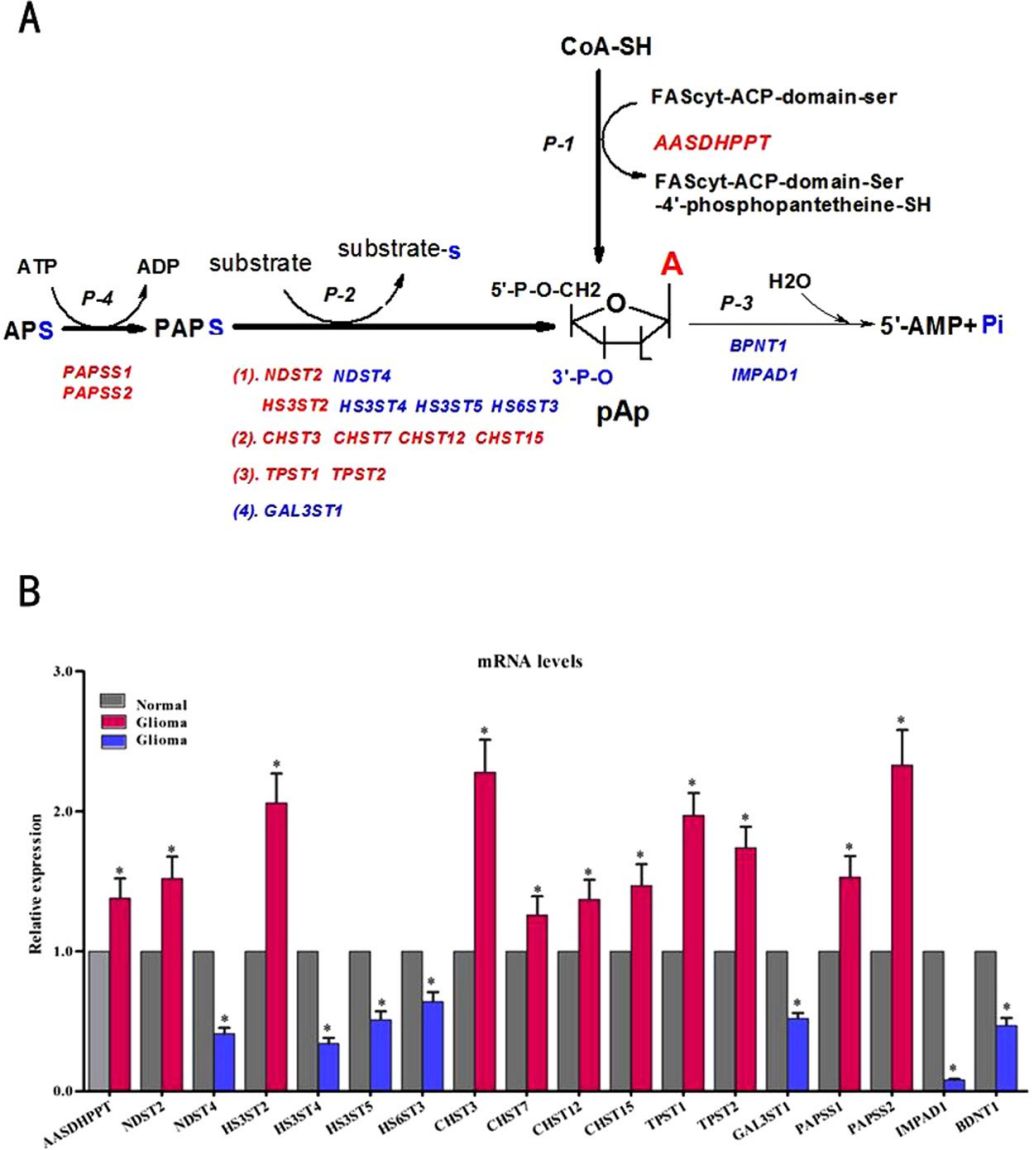
The gene expression profiling of 18 enzymes associated with pAp metabolism. (**A**) Catalytic reaction pathways of 18 enzymes. (**B**) Comparison of the relative expression level of each individual enzyme gene, using *GAPDH* as internal control. Glioma tissues (n = 59) and control brain tissues (n = 20) of discovery set and validation set were analyzed. Bars and gene names colored red represent the up-regulated genes, while blue bars and gene names represent the down-regulated genes, gray bars represent gene expression of the paired control. *p  < 0.05. (From *AASDHPPT* to *BNDT1*, the p value is 0.038, 0.032, 0.027, 0.036, 0.026, 0.019, 0.042, 0.018, 0.037, 0.032, 0.028, 0.017, 0.019, 0.034, 0.041, 0.015, 0.0067, 0.034; the fold change Mean ± SD is 1.38 ± 0.113, 1.52 ± 0.123, 0.41 ± 0.032, 2.06 ± 0.176, 0.34 ± 0.020, 0.51 ± 0.032, 0.64 ± 0.027, 2.28 ± 0.182, 1.26 ± 0.098, 1.37 ± 0.126, 1.47 ± 0.116, 1.97 ± 0.143, 1.74 ± 0.126, 0.52 ± 0.043, 1.53 ± 0.122, 2.33 ± 0.203, 0.08 ± 0.003, 0.47 ± 0.023, respectively). Abbreviation: FAScyt: cytosolic fatty acid syntha, other abbreviations refer to Table 3. Catalytic pathways of 18 enzymes: 1. P-1: activation pathway of FACcyt in fatty acid synthesis, P-2: sulphonation reaction pathway, P-3: pAp hydrolysis pathway and P-4: PAPS synthesis pathway. 2. Human genome encodes one phosphopantetheine transferase (*AASDHPPT)*, two 3′-nucleotidases (*IMPAD1* and *BPNT-1*), two PAPS synthase (*PAPSS1* and *PAPSS2*) and a number of sulfotransferases (*SULT*). A total of 13 sulfotransferases divided into 4 types according to substrates and products of enzymes. Refer to Table 3.

The gene expression profiling of 18 enzymes (using GAPDH as internal control) are shown in Fig. 4.

In the glioma tissues, the gene expression levels of *AASDHPPT*, *NDST2*, *HS3ST2*, *CHST3*, *CHST7*, *CHST12*, *CHST15*, *TPST1*, *TPST2*, *PAPSS1* and *PAPSS2* were significantly up-regulated (p < 0.05, Fig. 4). In contrast, the expression of *NDST4*, *HS3ST4*,*HS3ST5*,*HS6ST3* and *GAL3ST1* were down-regulated (p < 0.05, Fig. 4). Finally, we observed that gene expression levels of *IMPAD1* and *BPNT1* were significantly decreased in glioma tissues (p < 0.05, Fig. 4). *IMPAD1* and *BPNT1* enzymes are known to hydrolyze pAp to 5′-AMP and Pi^18^.

The change trend of gene expression profiling of 18 enzymes using TBP as internal controls was consistent with that of using GAPDH (Supplementary Figure, SF and Fig. 4).

## Discussion

Glioma is a common malignant brain tumor (1). To date, although many potential biomarkers have been found, there are still no effective therapeutic or diagnostic targets in clinical practice^9,19–21^.

This study revealed obvious variation of 8 novel phospholipids in glioma and identified their precise molecular structures (Tables 1 and 2). The contents of glycerophosphatidylcholine [PC(38:4), PC(36:3), PC(38:6)] and all lysoglycerophospholipids [lyso-PC(18:0), lyso-PE(18:3), lyso-PE(20:3) and lyso-PE(18:0)] in glioma were higher than that in control samples. On the contrary, glycerophosphatidylcholine PC(36:1) was not detected in glioma tissues, but was abundant in the control brain parenchyma. Limited information is avaiblable for the aberrant phospholipid metabolisms in glioma tissues^19,22^. Gliomas harboring the *IDH1-*R132H mutation demonstrated altered phospholipid metabolism characterized by decreased phosphoethanolamine levels and increased glycerophosphocholine levels^19^. However, Pavithra Viswanath demonstrated both phosphoethanolamine and phosphocholine levels were reduced in IDHmut glioma cells and in IDHmut gliomas in orthotopic tumor xenografts^14^. Jarmusch *et al*. using high-resolution desorption electrospray ionization method, demonstrated that gliomas has increased abundance of m/z794 [chloride adduct of PC(34:1)] and m/z885 [phosphatidylinositol (PI(18:0)/20:4)]^22^. A clinical study revealed that a elevated phosphocholine/glycerophosphocholine (PCho/GPC) ratio could be a negative predictive marker for bevacizumab efficacy^23^. Those gliomas may represent a malignant phenotype that could resist the anti-VEGF treatment. Therefore, the metabolic changes of phospholipid metabolites can serve as useful biomarkers.

The PC(36:1) was a very abundant composition in control brain parenchyma, which consists of one chain of vaccenic acid at the C-1 position and one chain of stearic acid at the C-2 position (HMDB 2018). It is a key component of the lipid bilayer of cells, as well as being involved in metabolism and signaling. PC (36:1) also is a common component in the plasma^24^. Therefore, the “complete” absence of PC(36:1) in glioma tissues is indeed a noticeable abnormality. The experiment result did not demonstrate a relationship between PC(36:1) absence and the glioma grade, it seems that PC(36:1) absence could result from a specific “mutation” or specific metabolic reprogramming that occurs in gliomas. However, the exact mechanisms of PC(36:1) absence in glioma tissues need to be explored in future studies.

In this study we observed that the content of DG(33:3) was increased in glioma. The analysis of neutral glycerolipid content proved useful in distinguishing gliomas from metastatic brain tumors by magnetic resonance spectroscopy (MRS)^25^. Chemically, eight lipid categories are reported, namely, fatty acyls (FA), glycerolipids, saccharolipids, polyketides, sterol lipids, prenol lipids, sphingolipids and phospholipids (PLs)^15^. Our experiment demonstrated that 5 lipid categories (13 lipids species), namely, glycerolipids, prenol lipids, cholesterol lipids, phospholipids (including PC and PE), sphingolipids (Ceramide and Sphingomyelin) were abnormal in glioma tissues. Therefore, it is plausible to speculate that abnormal metabolism of lipids could be a metabolomic phenotype in molecular pathology of glioma.

Lipidomics is an exciting new area^26^. It has been widely applied in several disciplines, including neuroblastoma^27^, mammalian cancer^28^, prostate cancer^29^, tetrahydrocannabinol addiction^30^, Alzheimer’s Disease^31^ and drug development^32^.

The experiment revealed that the level of pAp was significantly increased in the examined gliomas, whereas the content of pAp was very low in the control brain parenchyma. The VIP and fold change of pAp content were 7.12 and more than 500 times, respectively (Fig. 2, Table 2). pAp is a toxic by-product. It is a strong inhibitor for many enzymes. pAp inhibits PARP-1 [poly-(ADP-ribose) polymerase-1], which in turn results in abolished DNA repair, destruction of the chromatin structure, alteration in DNA methylation and cell death^17,33^. pAp can also inhibit nucleoside diphosphate kinase and oligoribonucleases. Deficiency of nuclease function results in the accumulation of immature ribosomal 5.8S RNA, which leads to the impairment of ribosome biogenesis^34,35^.

In mammalian cells, pAp primarily produced by the transfer of a sulfate group from PAPS to various acceptor molecules. This sulfate transfer reaction is catalyzed by a number of sulfotransferases (p-2 in Fig. 4). Apart from sulfur metabolism, pAp can also be generated during the transfer of the 4-phosphopantetheine group from CoA to acyl carrier protein of FAS, catalyzed by *AASDHPPT* protein in fatty acid synthesis^17^ (p-1 in Fig. 4). pAp is recycled into AMP and Pi by 3′-Nucleotidases^18,36,37^ (p-3 in Fig. 4). The exact mechanism of pAp level increase in glioma tissues is not clear till now. Therefore, we carried out the gene expressions of 18 enzymes associated with pAp metabolism to uncover the cause at transcription level. The up-regulation of phosphopantetheine transferase (*AASDHPPT*) and sulfotransferases (*NDST2*, *HS3ST2*, *CHST3*, *CHST7*, *CHST12*, *CHST1*, *TPST1* and *TPST2*) were directly responsible for the increase of pAp production. In humans both *PAPSS1* and *PAPSS2* possess ATP sulfurylase and APS kinase activity to synthesize PAPS^38,39^. As an active sulfate donor, PAPS is required by all sulfotransferases^39^. Therefore, *PAPSS-1* and *PAPSS-2* provided abundant PAPS substrate for sulfotransferases which leads to the increase of pAp production in gliomas.

The gene expressions of *IMPAD-1 (gPAPP*) and *BPNT-1* were remarkably down-regulated in glioma, especially the former (more than 90% reduction). *IMPAD-1* hydrolyzes the Golgi pAp yielding AMP and phosphate, *BPNT-1* hydrolyzes cytoplasm pAp to AMP and phosphate^18,36,37^. Therefore, it is reasonable to speculate that the deficiencies of *IMPAD-1* and *BPNT-1* function would lead to a lower degradation rate of pAp and hence promote its accumulation in glioma. Although *Bpnt-1* and *IMPAD-1* share a common substrate, their distinct subcellular localization suggests that they play unique roles in the cell^18,37^. Loss of *BPNT-1* function results in liver failure,whole-body edema and death, while the levels of bisphosphorylated nucleotides, pAp and PAPS, were dramatically elevated in the *BPNT-1* mutant liver and neurons^18,36^. Data showed that both gene expression of *IMPAD1* and *Bpnt-1* exist in human brain, the relative gene expression level of *IMPAD1* is higher than that of *BPNT-1* in human brain tissues (uniPROT 2018). Inhibition of *BPNT-1* causes selective neuronal dysfunction^36^. *IMPAD1* inactivation in mice and human produces neonatal lethality, lung abnormalities resembling atelectasis, dwarfism or chondrodysplasia with abnormal joint formation and impaired proteoglycan sulfation^18,36,37,40^. A recent study demonstrated that deletion of *BPNT-1* leads to accumulation of pAp, causing iron deficiency anemia^41^. To sum up, pAp accumulation may be a cause for molecular pathology of glioma.

Finally, the content differences of metabolites samples with different WHO grades did not observed. We speculated that the lacking difference maybe relate to specimen location in glioma tissues. The specimen pieces were commonly taken from the area without necrosis or hemorrhage of glioma in order to obtain histology diagnosis, whereas necrosis or hemorrhage is general characteristics of high -grade glioma. Further, this observation needs more case researches in the future.

## Conclusion

PC(36:1) absence and pAp accumulation were the most obvious metabolic abnormality in glioma. The gene expressions I*MPAD-1* and *BPNT-1* were dramatic down-regulated, which were the most primary causes for the pAp accumulation. PC(36:1) absence and pAp accumulation can be new clues to explore the molecular pathogenesis of glioma, further they may be used as potential therapeutic targets or biomarkers. Our findings suggest that abnormal metabolism of lipids and nucleotides play roles in the pathogenesis of glioma. To the best of our knowledge, this is the first study that reports the PC(36:1) absence and pAp accumulation in human glioma tissues.

## Methods

### Participant selection

59 adult glioma patients and 20 control subjects were enrolled in this study. All of participants did not suffer hepatic disease, adiposis and endocrine disease (diabetes, thyroid disease, adrenal cortex disease and so on).

The discovery set included 13 control specimens (F/M:8/5; age range: 23–71; average age: 50.6 ± 16.9), 9 WHO grade II glioma specimens (F/M: 4/5; age range: 27–63; average age: 48.5 ± 12.4) and 24 WHO grade III-IV glioma specimens (F/M: 13/11; age range: 32–75; average age: 48.9 ± 13.7). The validation set included 7 control brain parenchyma samples (F/M: 4/3; age range: 24–62; average age: 46.6 ± 13.3), 6 WHO grade II (F/M: 4/2; age range: 22–56; average age: 44.2 ± 12.5) and 20 WHO grade III-IV glioma samples(F/M: 8/12; age range: 26–63; average age: 45.3 ± 13.5). The demographic characteristics of patients and pathological diagnosis of specimens were mentioned in Supplementary Table ST. Statistical analysis demonstrated that there were no significant differences between discovery set and validation set (p > 0.05).

Tumor samples were obtained from gliomas during craniotomy. Gliomas were diagnosed and graded according to the WHO classification system. Control brain tissues were obtained from grossly normal brains of control subjects undergoing various surgical procedures that included cerebral injury and cerebral hemorrhage. Tissue specimens for the metabolomics study were immediately placed in cryogenic vials and snap-frozen in liquid nitrogen until component extractions. Other portions of the glioma and control brain tissues were stored in liquid nitrogen until RNA analysis.

The protocol for this study was revised and approved by the ethics committee of the First Hospital of Jilin University, Changchun, China. A written informed consent was obtained from all participants. All experimental procedures were carried out in accordance with the approved guidelines.

### Sample preparation of polar and non-polar solvent extracts

A two-step extraction procedure was carried out as described previously with some modification^42^. A total of 1500 μL chilled dichloromethane-methanol (3:1, v/v) was homogenized with 50 mg brain tissue in an ice-bath with a homogenizer (IKA, Staufen, Germany) for 30 seconds. The resulting homogenate was centrifuged at 12000 rpm for 25 minutes at 4 °C. Next, the supernatant was transferred to a fresh tube and used as the non-polar solvent extract. The residue was homogenized with 1500 μL methanol-water (1:1, v/v) as mentioned above, the supernatant was transferred to another fresh tube and used as the polar solvent extract. Subsequently, both fractions were dried under nitrogen, and stored at −80 °C until liquid chromatography/mass spectrometry (LC/MS) analysis.

### Non-targeted metabolomics analysis by UPLC-Q-TOF/MS

#### UPLC-Q-TOF/MS parameter

Chromatographic separation was performed on an Acquity ultra performance liquid chromatography (UPLC) Bridged Ethyl Hybrid (BEH) C18 column (2.1 mm × 100 mm, 1.7 μm, Waters Corp., Milford, USA) using Waters Acquity^TM^ UPLC system. The dried non-polar and polar solvent extracts was respectively dissolved in 500 μL of acetonitrile-water (1:1, v/v) and 500 μL of methanol-water (1:1, v/v), then combined, centrifuged at 13000 rpm for 20 min at 4 °C and 5 μL of the resulting mixture was injected into the UPLC system for analysis by BEH C18 column. The column was maintained at 40 °C and eluted at a flow rate of 0.45 mL/min, using a mobile phase of (A) 5% acetonitrile in water (by volume) and (B) 95% acetonitrile in water (by volume). The gradient program was optimized as follows: 0–1 min, 10% B to 50% B; 1–6 min, 50% B to 95% B; 6–8 min, 95% B to 98% B; 8–12 min, 98% B to 100% B and a final 12–14 min equilibration with 10% B. The eluent from the column was directed to the mass spectrometer without splitting.

#### Mass Spectrometry parameter

A Waters SYNAPT G2 HDMS (Waters Corp., Manchester, UK) was used to perform the mass spectrometry with an electrospray ionization source operating in a positive ion mode. The capillary voltage was set to 3.0 kV. Sample cone voltage and extraction cone voltage were set at 40 V and 4 V, respectively. Using drying nitrogen gas, the desolvation gas flow rate was set at 800 L/h at 450 °C, the cone gas rate was set at 50 L/h, and the source temperature was 120 °C. The scan time and inter scan delay were set at 0.3 s and 0.02 s, respectively. Leucine-enkephalin was used as the lockmass in a positive ion mode (*m/z* 556.2771[M + H] ^+^). Data was collected in centroid mode from *m/z* 50–1200 Da.

#### RNA Isolation and qRT-PCR analysis

The total RNA was extracted from 50 mg tissues using TRIzol Reagent (Invitrogen) according to the manufacturer protocol. RNA integrity was analyzed on 1.0% agarose gel and the quantity was determined using a NanoDrop 2000C Spectrophotometer (Thermo Scientific) according to the standard protocols. Subsequently, 1 ug RNA was reverse-transcribed with a PrimeScript™ RT reagent Kit (TaKaRa) for cDNA synthesis and genomic DNA removal was performed in accordance with the manufacturer’s protocols. The qPCRs were performed in triplicate sets with a SYBR premix Ex Taq™ II kit (Tli RNaseH Plus; TaKaRa) in a real-time PCR detection system (TaKaRa) according to the provided instructions. Gene-specific primers were designed using primer-blast (http://www.ncbi.nlm.nih.gov/tools/primer-blast/). The primer sequences were listed in Table 4. Glyceraldehyde-3-phosphate dehydrogenase (*GAPDH*) and TATA-box binding protein (*TBP*) were selected as endogenous controls. The primer sequences are as follows: *GAPDH*(138 bp), F: GCACCGTCAAGGCTGAGAAC, R: TGGTGAAGACGCCAGTGGA; *TBP* (132 bp), F: TGCACAGGAGCCAAGAGTGAA, R: CACATCACAGCTCCCCACCA. The specificity of amplification was assessed by dissociation curve analysis, and the relative abundance of genes was determined using the 2^−ΔΔCt^ method. The mRNA expression levels of 18 genes were compared between glioma tissues (discovery set and validation set, n = 59) and normal brain tissues (discovery set and validation set, n = 20), and all data were expressed as Mean ± standard deviation. The means between the two groups were compared using Student’s t-test and p < 0.05 was considered statistically significant.

**Table 4:** Primer sequences of the 18 enzymes related to pAp metabolism.

NO	Gene		Primer Sequence (5′->3′)	NO	Gene		Primer Sequence (5′->3′)
1	AASDHPPT	F	CAGTGCTTGCTGCTGAACCTGAG	10	CHST12	F	TCTTGTTCACTCCACTGCCTCTATC
		R	GCTTCTGATTGTTTCCCATTCTTTG			R	ATTTTACTCCAGCATTCCCTTCC
2	NDST2	F	GTCCTCCGGGTATGAAGCTAAAA	11	CHST15	F	GAGGACTGAAGGGAACGAAAACTG
		R	CCTCACATCAAAGGTCAGCAAAG			R	CCGTAATGGAAAGGTGATGAGATC
3	NDST4	F	CAGGGACTGAAGAGGAAGATGAA	12	TPST1	F	GGCATGTCTGGTGATTAGTTCTGT
		R	ATGACTCGTTGTGGAAGAGGTGG			R	AGGTTTTGTTGGCTTTGAGGTCC
4	HS3ST2	F	CTGGAGTTTATCCGAGTACACCCG	13	TPST2	F	ATGGAGGTAGGCAAGGAGAAGTG
		R	GGAGCCTCTTGAGTGACAAAGTAGC			R	AAGTCGAGGATGAGCTTGAGTGA
5	HS3ST4	F	GCTCATCATCGGGGTCAAGAAAG	14	GAL3ST1	F	TTGTTCGAGTCCTCCTTCCACTA
		R	CATCCAAAGTCTTGGGCATCACA			R	GTCTTGCAGGAACTCGGTCAGC
6	HS3ST5	F	CTGCTTGAAATGCTGAACCTACA	15	PAPSS1	F	AACAGATGCGGAAACATTACCAG
		R	ACCACTCAATGCCCTTACCATAA			R	CACCTCCATCCAGAAGACAATCA
7	HS6ST3	F	TCTCACTGTGCTTGGCTTCATC	16	PAPSS2	F	GAACCAGCAGAAATCCACCAATG
		R	CTGTGGGCTTCACTATTTTCATCTA			R	CTCCTCCAGGGCAAAACTTATCG
8	CHST3	F	GGTTGGCTGTGCTGGTTACTGAT	17	IMPAD1	F	ACAGGTCGCTCTTCAGACTTTTG
		R	TGTGCCTTTATGATGCTTGTCTATG			R	TCTTGACTCTTATCAGGCACATCC
9	CHST7	F	CTTGCTACCAACAACTGCTGTGC	18	BPNT1	F	ACGAGGGACCTGGGTCTACG
		R	ACTTCATCTCAACCAATGCGTCC			R	GGAGGCTACCAACCGCATCA

### Statistical analysis

#### Multivariate statistical analysis

The raw spectral data were analyzed with MassLynx Applications Manager Version 4.1 (Waters, Manchester, UK). Deconvolution, alignment and data reduction were performed to generate a list of retention times (RT) and mass pairs with a corresponding peak area for all the detected peaks from each file in the data set. The main parameters were set as following: RT range 0.5–14 min; mass range 50–1200; extracted ion chromatograms (XIC) at 0.02 Da mass window; automatic calculation of the peak width and peak–peak base-line noise; the use of raw data during the deconvolution procedure; marker intensity threshold (count) was set at 300; the mass tolerance was 0.02 Da; RT windows were 0.2 s; the noise elimination level was set at 6 to retain the isotopic peaks. The resulting UPLC-MS data were then transferred to SIMCA-P software package (version 12.0, Umetric, Umeå, Sweden). Principal component analysis (PCA) analysis was performed by the Markerlynx 4.1 software to discern the metabolic profiles of glioma and control tissues and the results were determined by the visual inspection of score plots. Supervised models were subsequently performed by orthogonal partial least squares discriminant analysis (OPLS-DA) to maximize the separation between the different classes and identify biomarkers associated with glioma. The results were visualized in the form of score plots and the potential biomarkers were selected based on the variable importance in the project (VIP) value and S-plot.

#### Mathematical statistics

Statistical analyses were performed using the statistical software package (SPSS for Windows, version 21.0; IBM-SPSS, Chicago, IL, USA). Box plots were performed using MetaboAnalyst 3.0^43^. The distribution of continuous variables was assessed using the Kolmogorov-Smirnov test. Normally distributed variables were presented as mean ± standard deviation (SD). Non-normally distributed variables were presented as median (IQR). Data were plotted as mean ± standard error of the mean (SEM). Kruskal-Wallistest, one-way ANOVA and independent sample *t* test were performed to determine the difference between the examined groups. A *P value *<* 0*.05 was considered to statistically significant.

### Ethics approval and consent to participate

This study was reviewed and approved by the Ethics Committee of First Hospital of JiLin University, and all patients provided written informed consent. All experimental procedures were carried out in accordance with the approved guidelines.

## Electronic supplementary material

supplementary information

## Data Availability

The authors declare that datasets supporting the conclusions of this study are available within the manuscript and its supplementary information files.
