# Supplementary material for: Glycerophosphatidylcholine PC(36:1) absence and 3′-phosphoadenylate (pAp) accumulation are hallmarks of the human glioma metabolome
Source: Sci Rep. 2018 Oct 3;8:14783. doi: 10.1038/s41598-018-32847-8 (PMC6170378; doi:10.1038/s41598-018-32847-8)
Supplement: Supplementary file 1 — supplementary information [file 41598_2018_32847_MOESM1_ESM.docx]

**Supplementary File**

**Glycerophosphatidylcholine PC(36:1) absence and 3'-phosphoadenylate（pAp） accumulation are hallmarks of the human glioma metabolome**

Wenchen Li^1^**^#^ ,** Hongmei Jia^2^**^#^**  , Qi Li^3^, Jiayue Cui^4^, Ri Li^1^， Zhongmei Zou^2*+^, Xinyu Hong ^1*+^

^1^ Department of Neurosurgical Oncology, The First Hospital of Jilin University, Changchun, PC:130021,China.

^2^ Institute of Medicinal Plant Development, Chinese Academy of Medical Sciences and Peking Union Medical College, Beijing, PC:100191, China

^3^ Core Laboratory for Clinical Medical Research, Beijing Tian Tan Hospital, Capital Medical University, Beijing, PC:100050 China

^4^ Department of Histology and Embryology of Basic Medicine College, Jilin University, Changchun, PC:130021, China.

^*+^ or **^#^** : These authors contributed equally to this work.

**Corresponding authors:**

**Zhongmei Zou^*+^ :** zmzou@implad.ac.cn

**Xinyu Hong^*+^ :** hongxy@jlu.edu.cn

**Supplementary Table（ST）：**

**Demographic Characteristics of Patients and Pathological Diagnosis of Specimens**

|  | **Discovery set** | | |  | **Validation set** | | | | |
| --- | --- | --- | --- | --- | --- | --- | --- | --- | --- |
|  | **III-IV Grade** | | |  | **III-IV Grade** | | | | |
| No | Gender | Age | pathological diagnosis | No | Gender | Age | | pathological diagnosis | |
| 1 | M | 63 | R Tem glioblastoma | 1 | F | 30 | | R Tem anaplastic oligoastrocytoma | |
| 2 | F | 64 | L Par glioblastoma | 2 | F | 28 | | R Tem glioblastoma | |
| 3 | M | 49 | R Tem anaplastic astrocytoma | 3 | M | 58 | | L Tem  glioblastoma | |
| 4 | F | 32 | R Tem anaplastic oligodendroglioma | 4 | M | 26 | | L Fro anaplastic oligoastrocytoma | |
| 5 | M | 45 | R Fro glioblastoma | 5 | M | 46 | | R Fro glioblastoma | |
| 6 | F | 40 | Corpus callosum  glioblastoma | 6 | F | 47 | | R Tem anaplastic oligoastrocytoma | |
| 7 | M | 48 | L Fro glioblastoma | 7 | F | 27 | | L Tem anaplastic oligodendroglioma | |
| 8 | F | 32 | L Tem glioblastoma | 8 | M | 55 | | R Tem glioblastoma | |
| 9 | F | 66 | L Fro glioblastoma | 9 | M | 27 | | R Tem glioblastoma | |
| 10 | M | 60 | L Tem glioblastoma | 10 | M | 50 | | L Fro glioblastoma | |
| 11 | M | 37 | R Tem anaplastic oligodendroglioma | 11 | M | 39 | | R Tem glioblastoma | |
| 12 | M | 42 | L Fro glioblastoma | 12 | F | 51 | | R Fro glioblastoma | |
| 13 | M | 75 | L Tem glioblastoma | 13 | M | 61 | | R Tem glioblastoma | |
| 14 | F | 37 | R Tem  anaplastic astrocytoma | 14 | M | 51 | | L Fro anaplastic oligoastrocytoma | |
| 15 | F | 64 | L Tem glioblastoma | 15 | M | 33 | | L Ven anaplastic ependymoma | |
| 16 | M | 57 | L Fro and Tem  glioblastoma | 16 | F | 54 | | L Fro glioblastoma | |
| 17 | F | 32 | L Fro and Tem anaplastic glioblastoma | 17 | F | 63 | | Corpus callosum  glioblastoma | |
| 18 | M | 37 | L Fro glioblastoma | 18 | M | 35 | | L Tem glioblastoma | |
| 19 | F | 32 | L Tem glioblastoma | 19 | M | 62 | | L Tem glioblastoma | |
| 20 | F | 58 | L thalamus  glioblastoma | 20 | F | 63 | | R. Fro glioblastoma | |
| 21 | F | 68 | R Tem glioblastoma |  |  |  | |  | |
| 22 | M | 56 | L Fro glioblastoma |  |  |  | |  | |
| 23 | F | 46 | R Par glioblastoma |  |  |  | |  | |
| 24 | F | 33 | R Fro gliosarcoma |  |  |  | |  | |
| No | **II Grade** | | | No | **II Grade** | | | | |
| 1 | F | 51 | R Tem oligodendroglioma | 1 | F | 39 | | | R Fro diffuse astrocytoma |
| 2 | M | 60 | L Fro oligoastrocytoma | 2 | F | 55 | | | R Fro oligoastrocytoma |
| 3 | M | 50 | L Fro oligodendroglioma | 3 | M | 56 | | | R Fro oligoastrocytoma |
| 4 | F | 30 | L Fro pilomyxoid astrocytoma | 4 | F | 46 | | | R Tem oligoastrocytoma |
| 5 | F | 63 | L Tem diffuse astrocytoma | 5 | F | 47 | | | R Tem oligodendroglioma |
| 6 | M | 50 | L Tem  diffuse astrocytoma | 6 | M | 22 | | | L Occ oligoastrocytoma |
| 7 | M | 27 | L ventricle central neurocytoma |  |  |  | | |  |
| 8 | M | 57 | L Par diffuse astrocytoma |  |  |  | | |  |
| 9 | F | 49 | L Fro oligoastrocytoma |  |  |  | | |  |
| No | **Control Group** | | | No | **Control Group** | | | | |
| 1 | M | 37 | R Tem  brain trauma | 1 | F | | 62 | | R Tem  brain hemorrhage |
| 2 | M | 48 | L Tem  brain hemorrhage | 2 | M | | 40 | | L Tem  brain trauma |
| 3 | F | 69 | L Tem  brain hemorrhage | 3 | F | | 41 | | R Tem  brain hemorrhage |
| 4 | F | 24 | L Fro brain trauma | 4 | F | | 49 | | R Tem  brain hemorrhage |
| 5 | M | 49 | L Fro brain trauma | 5 | F | | 48 | | R Fro brain trauma |
| 6 | F | 54 | L Tem brain trauma | 6 | M | | 62 | | L Fro brain trauma |
| 7 | F | 59 | R Tem brain hemorrhage | 7 | M | | 24 | | L Fro brain trauma |
| 8 | F | 71 | R Fro brain trauma |  |  | |  | |  |
| 9 | F | 23 | L Fro brain trauma |  |  | |  | |  |
| 10 | F | 36 | L Tem brain trauma |  |  | |  | |  |
| 11 | M | 69 | R Tem brain hemorrhage |  |  | |  | |  |
| 12 | M | 49 | L Tem brain trauma |  |  | |  | |  |
| 13 | F | 70 | R Fro brain hemorrhage |  |  | |  | |  |

Abbreviation : M:male, F:femal, L: left, R: right, Tem: temporal lobe, Fro: frontal lobe , Par: parietal lobe, Occ: occipital lobe， Ven: ventricle

**Supplementary Figure(SF)：**


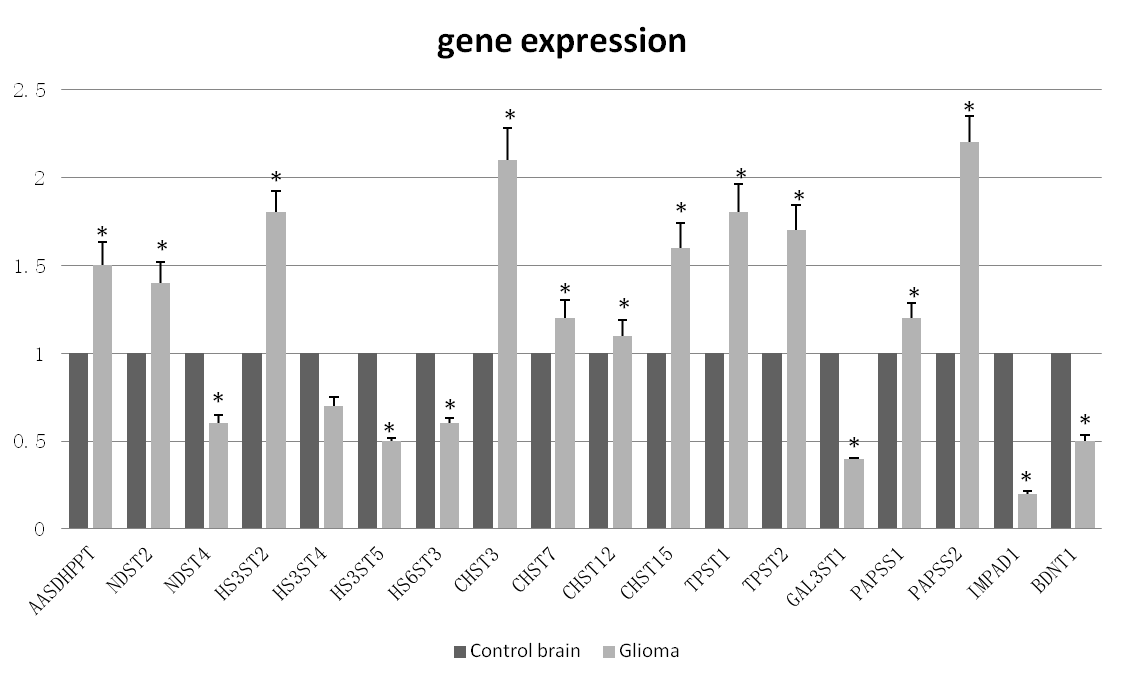


**SF：**The gene expression profiling of 18 enzymes associated with pAp metabolism using TATA-box binding protein (*TBP*) as an endogenous control.

Glioma tissues (n=59) and control brain tissues (n=20) of discovery set and validation set were analyzed. The change trend of gene expression profiling of 18 enzymes using *TBP* as internal control was consistent with that of using *GAPDH* (Fig.4).

These 2 genes represent different physiological pathways. *GAPDH* controls glycolysis and gluconeogenesis in carbohydrate metabolic pathway, while TBP is RNA polymerase II, a transcription factor, which regulates transcription process.
